# Sex Steroids Effects on Asthma: A Network Perspective of Immune and Airway Cells

**DOI:** 10.3390/cells11142238

**Published:** 2022-07-19

**Authors:** Niyati A. Borkar, Colin Kelly Combs, Venkatachalem Sathish

**Affiliations:** 1 Department of Pharmaceutical Sciences, School of Pharmacy, College of Health Professions, North Dakota State University, Fargo, ND 58102, USA; niyatiarun.borkar@ndsu.edu; 2 Department of Biomedical Sciences, School of Medicine & Health Sciences, University of North Dakota, Grand Forks, ND 58202, USA; colin.combs@und.edu

**Keywords:** asthma, innate immunity, adaptive immunity, estrogen, testosterone, kisspeptin

## Abstract

A multitude of evidence has suggested the differential incidence, prevalence and severity of asthma between males and females. A compilation of recent literature recognized sex differences as a significant non-modifiable risk factor in asthma pathogenesis. Understanding the cellular and mechanistic basis of sex differences remains complex and the pivotal point of this ever elusive quest, which remains to be clarified in the current scenario. Sex steroids are an integral part of human development and evolution while also playing a critical role in the conditioning of the immune system and thereby influencing the function of peripheral organs. Classical perspectives suggest a pre-defined effect of sex steroids, generalizing estrogens popularly under the “estrogen paradox” due to conflicting reports associating estrogen with a pro- and anti-inflammatory role. On the other hand, androgens are classified as “anti-inflammatory,” serving a protective role in mitigating inflammation. Although considered mainstream and simplistic, this observation remains valid for numerous reasons, as elaborated in the current review. Women appear immune-favored with stronger and more responsive immune elements than men. However, the remarkable female predominance of diverse autoimmune and allergic diseases contradicts this observation suggesting that hormonal differences between the sexes might modulate the normal and dysfunctional regulation of the immune system. This review illustrates the potential relationship between key elements of the immune cell system and their interplay with sex steroids, relevant to structural cells in the pathophysiology of asthma and many other lung diseases. Here, we discuss established and emerging paradigms in the clarification of observed sex differences in asthma in the context of the immune system, which will deepen our understanding of asthma etiopathology.

## 1. Introduction

Asthma is a common chronic respiratory disease afflicting more than 300 million people worldwide [1]. Hallmark asthma symptoms include chest tightness, shortness of breath, and coughing, all of which fluctuate over time, leading to increased respiratory distress and worsening symptoms, characteristic features of severe asthmatics [2,3,4,5,6,7]. Asthma etiology revolves around classical features of an immune response, primarily regulated and maintained by the persistent activation of chronically activated T-cells [8,9]. Asthma has long been considered a T helper type 2 cell (Th2)-mediated process concurrent with multiple studies supporting the Th2-driven hypothesis. Gradually, additional work highlighting the importance of other immune cells such as basophils, mast cells and eosinophils, which can further contribute to the Th2-associated cytokine drift in asthma, were reported, suggesting “Th2-cell-high” and the “Th2-cell-low” categories of asthma [10]. Thus, although most airway damage observed in asthma results from persistent inflammation involving an array of immune cell elements, the consensus remains that T cells are central in the pathophysiology of asthma. Accordingly, multiple trials focused on T cell-associated molecular targets have been conducted to identify therapies against uncontrolled asthma [9,11,12].

Sex differences in asthma are influenced by the interplay between sex chromosomes, sex steroids and the immune system [13]. The female bias in disease expression is well established across many diseases, including asthma. For instance, the male predominance in asthma before puberty (from infancy to childhood) suggests a sex chromosome role in the incidence and prevalence during the early stages of life [14]. This suggests that the influence of sex chromosomes in disease incidence occurs independently of sex steroid effects. The four-core genotype (FCG) mouse model is a novel technique used widely to study the role of XX versus XY genes separately from the sex steroidal effects [15]. Moreover, in the case of females, factors such as menstrual cycle, pregnancy, use of oral contraceptives (OCPs), and menopause also exist. Women generally suffer greater respiratory discomfort and exacerbation of asthma symptoms during the menstrual cycle primarily due to fluctuations in the sex steroid levels throughout menses [16,17]. On the other hand, women on birth control pills or OCPs experience fewer asthma attacks and are at a lesser risk of developing asthma [18]. Interestingly, asthmatic symptoms worsen during pregnancy and vary in each trimester [19]. Menopausal females may experience worsened or improved asthmatic symptoms, adding more complexity to this scenario. This could be attributed to unbalanced levels of female sex steroids [20]. Overall, different factors, including epigenetic factors and hormonal mediators between males and females, have been accounted for in sex-based disparities observed in asthma. Notably, there is also considerable interest in understanding sex differences in allergic responses that influence the phenotype of T cells and asthma. It has been more than two decades since the evidence was published linking the interrelationship of sex steroids and immune responses in the pathobiology of asthma [13,14,15]. Clinical studies [16] and animal model work [17,18,19], including our own [20,21,22,23,24], support a direct relationship between sex steroid-mediated immune cell behavior and asthma severity. Here, we attempt to briefly review the updated role of sex steroids in regulating the immune system response of allergic asthma.

## 2. Airway Inflammation in Asthma

Airway inflammation, together with airway hyperresponsiveness (AHR) and airway structural remodeling, is one of the most noticeable features of asthma, a phenotypically heterogeneous respiratory disease that affects about 8% of the worldwide population and is still growing [6,25,26,27,28]. Clinically, bronchial biopsies from asthmatics show distinguishing structural changes with collagen deposition under the epithelium (also described as reticular basement-membrane thickening, RBM) and increased airway smooth muscle (ASM) as a result of hyperplasia and hypertrophy [27,29,30]. The spectrum of asthma severity varies from mild to moderate to severe uncontrolled disease depending on the inflammatory cell types involved. An immensely large array of lymphocytes, pro-inflammatory cytokines and chemokines released by immune and airway structural cells significantly contribute to the different asthma phenotypes. Typically, asthma is characterized by two major predominant phenotypes: eosinophilic vs. neutrophilic and T helper type 1 (Th1) vs. Th2, or even mixed eosinophilic/neutrophilic inflammatory patterns [27,31]. However, newer variants of the T-cell immune response, such as T helper type 9 (Th9), T helper type 17 (Th17) and T helper type 22 (Th22), have also been shown to contribute to immune cell responses in asthma [32,33]. Eosinophilic inflammation can be associated with the spectrum of asthma severity, ranging from mild to moderate to severe uncontrolled disease, whereas neutrophilic inflammation occurs typically in more severe asthma [34,35]. Eosinophilic asthma includes Th2 cell-derived cytokines that cause allergic or nonallergic phenotypes, while neutrophilic asthma is characterized by Th17 cell-inflammatory mechanisms [36]. The major difference between these types of asthma intensities is the radical and gradual amalgamation of the cytokines and chemokines produced by these immune cells. These chemokines cross-link with each other to propagate and intensify an allergenic or asthmatic attack. Although categorized as eosinophilic vs. neutrophilic attacks, other immune cells, such as those regulated by Th9, Th17, and Th22 have not been fully characterized. Concurrent with this hypothesis, another paradigm often overlooked is the influence of sex steroids on the immune system in asthma.

Classically, asthma is considered a sex-differentiated heterogeneous inflammatory disorder of the conducting airways that is strongly associated with an allergic reaction to barrier function followed by a defect in the immune response [37,38,39]. This allergenic sensitization repeats itself due to a lack of successful therapies leading to disease persistence, transpiring into “chronic or severe asthma”. Severe asthma is characterized by a polarized eosinophilic-driven Th2 mediated-immune response, exaggerated in the case of females [40]. However, some patients also exhibit a Th2-low phenotypic expression in combination with upregulated expression of non-Th2 cytokines [41]. An important characteristic of Th2-high asthma is that it is responsive to corticosteroids, whereas Th2-low asthma is not [42,43]. Neutrophilic and pauci-granulocytic cellularities are exhibited by Th2-low asthmatic patients [44,45]. Specific to neutrophilic asthma, a study confirmed the positive correlation between sputum IL-8 levels and neutrophilic airway inflammation [46]. Other studies reconfirmed that elevated levels of C-X-C Motif Chemokine Ligand 8 (CXCL8) or IL-8 and neutrophil elastase are important biomarkers of Th2-low asthma [47]. Unfortunately, targeting this disease with immune cell type selective therapies has proven to be extremely disappointing, necessitating a need to re-evaluate the therapeutic strategies used to modulate, if not treat, this disease. There are several hallmark characteristics of asthma: epithelial basement membrane thickening, an increased (hyper-proliferated) ASM mass [3,4,29,48,49], and enhanced numbers of fibroblasts [4,50]. Thus, what is seen in asthma as a single cellular and inflammatory response to allergens is more complex and involves a complicated interplay between the sex of an individual, sex steroid regulation characterized by estrogen vs. testosterone effects, the structural and formed elements of the airway and, last but not the least, the immune reactivity to these confounding factors. Therefore, this review will provide a comprehensive overview of the existing literature on immune cell dysregulation in asthma and the influence of sex steroids on immune function in the pathogenesis of asthma.

Epithelial membrane thickening and increased airway smooth muscle comprise the predominantly affected cell types in asthma [50,51,52]. Evidence suggests a critical role of the epithelium in producing mediators of inflammation by both immune cells and other cell types of the airways. The reported thickness of the RBM in asthmatic patients varies considerably. Normal RBM thickness values in healthy controls range from 3 to 7 µm, whereas measurements in asthmatics range from 7 to 23 µm [3,26]. The RBM thickness in asthma is determined by the proportion of continuous deposition and degradation of proteins such as collagen-I, collagen-III, collagen-V, fibronectin, tenascin, lumican and biglycan [3,29,51]. ASM cells modulate the conducting airways by secreting inflammatory mediators (e.g., TGF-β), extracellular matrix proteins, and expressing cell adhesion molecules and other costimulatory molecules involved in the migration and activation of inflammatory immune cells. Clinically, Th1 inflammation-associated cytokine, tumor necrosis factor-alpha (TNFα), has been proposed as a mainstay target of asthma therapy by depleting the contractile aspects of asthma [53]. However, how these processes can be utilized against asthma therapeutically is unclear and still under investigation among the different cell types in the lung.

## 3. Sex Steroids and Immune Function

The immune system is highly conserved across species and mainly consists of the innate immune system, which mounts a rapid response to infections by relying on pattern recognition unique to pathogens, and an adaptive immune system that provides antigen-specific responses, which are heightened at the time of infection [10,54,55]. Cells of the innate immune system comprise granulocytes (neutrophils, eosinophils and basophils), monocytes, macrophages, dendritic cells (DC), mast cells (MC) and natural killer (NK) cells [41,56]. The adaptive immune responses are based on specific antigen receptors on T- and B-lymphocytes [57]. Interactions between the innate and adaptive immune systems are largely mediated by cytokines, which coordinate the actions of different immune functions to efficiently target infections from various types of pathogens [30,31]. Depending on infectious triggers and the time of exposure to the antigen, the innate immune cells present the processed antigens to specialized cells of the adaptive immune system. The cell-mediated immune system consists of intracellular focal pathogens and two types of effector cells: CD8+ cytotoxic T-cells and CD4+ helper T-cells. CD8+ cytotoxic T-cells (or cytotoxic T-lymphocytes (CTLs)) eradicate infected host cells, and CD4+ helper T-cells stimulate macrophages to eliminate microbes. In humoral immunity, the B-cells (or B-lymphocytes) function as effector cells upon stimulation by CD4+ helper T-cells. B-cells produce antibodies that bind to and facilitate the removal of extracellular pathogens and toxins. Four main groups of CD4+ helper T-cells called Th1, Th2, Th17 cells, and T-regulatory cells (Treg) each initiate a specific immune response [8,9]. Classically, Th1-cells predominantly activate macrophages and CTLs, while Th2 cells mainly stimulate B-cells to produce antibodies and Th17 cells induce the production of inflammatory cytokines [58,59]. Treg cells are involved in the normal regulation of immune responses [60].

Sex steroids regulate reproductive and metabolic body functions throughout the lifespan of an individual [2,61]. In addition, sex steroids also influence immune cell function and disease conditions. Androgens are predominantly anti-inflammatory, whereas estrogens have pro- and anti-inflammatory roles, depending on numerous factors [62,63,64]. Therefore, mechanisms of sex-steroidal regulation of the immune system, notably on different cell types, are imperative to understand how a competent and healthy immune system is maintained. An important demonstration of this basic fact is the underlying differences in the activities of immune cell types in men vs. women, which are attributed to sex steroids [64,65]. Researchers and clinicians have observed striking differences between the immune responsiveness of males and females [64,66]. In general, females have superior humoral and cell-mediated immunity [67]. Simultaneously, clinicians have noted that women are more resistant to various infections, which correlates with their greater longevity [37,68]. Although these interpretations implicate the influence of sex-hormonal factors, sex steroid involvement in regulating pulmonary diseases such as asthma has received relatively scant attention.

**Figure 1:**
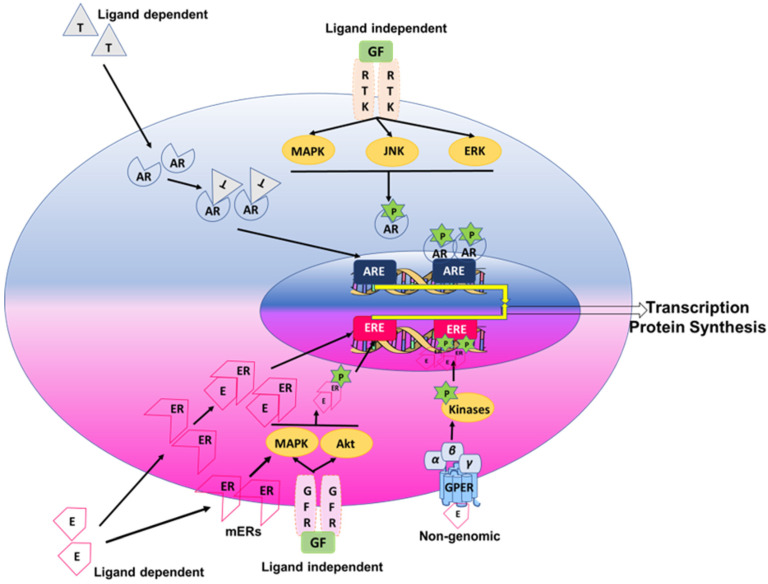
Schematic diagram illustrating the intracellular genomic (ligand-dependent and -independent) and non-genomic pathways of estrogen and testosterone. The genomic effects of sex steroids occur through their binding to cytoplasmic or membrane steroid receptors or through direct phosphorylation of other coregulators. Genomic signaling is a classic mechanism of action where phosphorylated hormone receptor complexes translocate to the nucleus and further bind to steroid receptor elements in the promoter regions of the target genes. Indirect genomic effects occur when sex steroids regulate gene expression by regulating other transcription factors or kinases. Abbreviations: E, estrogen; T, testosterone; AR, androgen receptor; ER, estrogen receptor; mER, membrane estrogen receptor; RTK, Receptor tyrosine kinases; GF, Growth Factors; ARE, androgen response elements; ERE, estrogen response elements; GFR, Growth factor receptor; GPER, G-protein estrogen receptor 30.

Testosterone or androgens via the androgen receptor (AR) have been postulated to have an overall immunosuppressive effect [69,70], while estrogen may generally have immunoenhancing effects [71]. The generalization is that testosterone favors Th1-type immune-profile diseases [72], while estrogen and progesterone favor Th2-type immune-profile diseases [73,74]. Sex steroids exert their effects by binding to their cognate intracellular receptors or transcription factor nuclear receptors. Estrogens acting via estrogen receptors (ERs: ERα and ERβ) exert both pro- and anti-inflammatory effects depending on the relative expression of ERα and ERβ, the target organ and its microenvironment, the concentration of the sex steroid and the type of the immune cell target [62,75,76]. Additionally, the signaling mechanisms of sex steroid receptors vary based on the outcome of their respective signaling cascades. An overview of the classical genomic and non-genomic signaling mechanisms for estrogen and testosterone with their receptors, namely ER and AR, is shown in Figure 1. ERs are expressed in various immune cells, such as lymphocytes, macrophages and dendritic cells [70,77]. Progesterone acting via the progesterone receptors (PRs: PR-A and PR-B) is expressed in immune cells, such as T cells, macrophages, mast cells, dendritic cells and natural killer cells [78,79,80,81]. Progesterone is known to have a diverse and broad anti-inflammatory effect on the immune system by decreasing pro-inflammatory mediators via inhibiting leukocyte activation [82]. Androgens, including testosterone and dihydrotestosterone (DHT), show immunosuppressive activities by promoting anti-inflammatory mediator release by T cells and macrophages [15,83]. While a great deal of research explains the possible discrepancy between sex-differentiated occurrences of diseases such as asthma, COPD, and pulmonary hypertension, a major question remains concerning the effects of sex steroids on the pulmonary immune system and their impact on the normal and disordered functions of the lung.

## 4. Biological Effects of Sex Steroids: Immune Cell Milieu in Asthma

**Figure 2:**
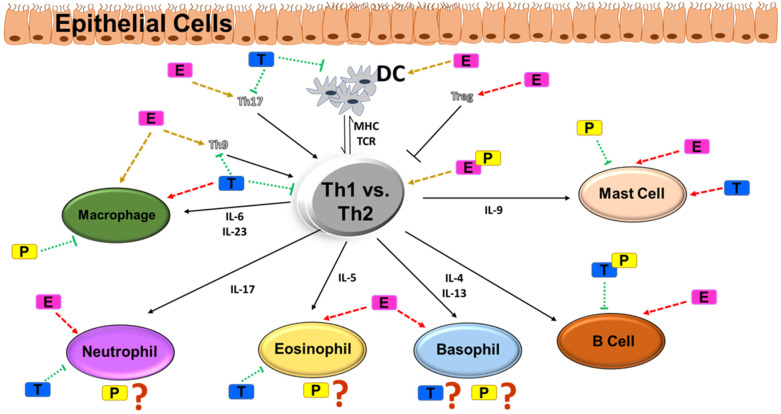
Th1 vs. Th2 type of inflammation and their effects in immune cells: modulatory role of sex steroids. Several studies have postulated a Th1 vs. Th2 driven hypothesis of airway inflammation and the critical role of immune cell types such as macrophages, neutrophils, eosinophils, basophils, B cells and mast cells. The normal functioning of these immune cell types is affected by circulating concentrations of estrogen vs. testosterone vs. progesterone, the effect influenced by the cyclical variations of the sex steroids. Pathways aggravated during asthma are marked by red arrows (dotted), whereas pathways inhibiting airway inflammation in asthma are marked by green arrows (dotted). Yellow arrows denote unknown effects. Abbreviations: DC, dendritic cells; MHC, major histocompatibility complex; TCR, T-cell receptor; IL, interleukin; E, estrogen; T, testosterone; P, progesterone.

According to the Expert Panel 2020 focused updates to the 2007 Asthma Management Guidelines by the National Heart, Lung and Blood Institute (NHLBI), immunohistopathologic features of asthma have been recognized as a major predisposing factor contributing to the development of asthma. As already mentioned, the pathophysiology of asthma involves a signaling cascade of changes occurring in several cell types. Although distinct in origin, these cells exhibit similar changes in critical function, thereby causing a synergistic effect on disease. Therefore, a careful scrutinization of sex steroid effects on each cell type is required to understand and discriminate the biology of asthma. A schematic overview of sex steroid effects on immune cells is depicted in Figure 2.

### 4.1. Sex Steroids and Basophils

Basophils, discovered by Paul Ehrlich in 1879, constitute the least abundant granulocyte population in the peripheral blood, comprising less than one percent of leukocytes, and not many studies have been carried out on basophils as a prime interest [84]. Therefore, they are the least investigated leucocyte/granulocyte as far as sex steroid effects are concerned. Histamine is a major mediator of allergy and inflammation in asthmatic patients, and studies have shown that estrogens promote increased histamine secretion from human basophils [77,85]. Another study investigating the effects of progesterone treatment during influenza virus showed increased numbers of immune cells and improved pulmonary function in the lungs of IAV-infected female mice via a repair of epithelial cells [86]. In a clinical study on the effects of testosterone on hematopoietic variables, it was shown that exogenous testosterone did not influence circulatory basophil count in male study participants [87]. However, more studies to deduce the role of testosterone on basophils in asthma and lung diseases are warranted.

### 4.2. Sex Steroids and Eosinophils

Eosinophils comprise one of the major immune cells recruited immediately after post-allergic inflammation [36,88]. Apart from their central role in allergic asthma, eosinophils perform various functions such as secreting various chemokines, cytokines and growth factors. Eosinophils are recruited by a signaling cascade initiated when an allergen interacts with the T-cell lymphocyte, which in turn effectively induces eosinophil activation/migration processes via the vascular cell adhesion molecule (VCAM) and the intercellular cell adhesion molecule (ICAM-1) [89]. Specific cytokines such as IL-5 and IL-13 stimulate eosinophil activation following this stimulation. IL-5 and IL-13 selectively stimulate eosinophils by influencing specific chemo-attractants such as eotaxin [89]. Aggravating this situation, locally produced IL-4 and IL-13 promote adhesion of eosinophils to the vasculature, further promoting the release of IL-5 and the growth factor, granulocyte-macrophage colony-stimulating factor (GM-CSF), which promote the survival of eosinophils. After that, a chain of events enumerates airway inflammation, including airway obstruction, injury to the airway walls and airway hyperresponsiveness, which all contribute to airway remodeling [89,90]. A point to note here is that asthma is categorically characterized by eosinophilic infiltration. Clinical evidence suggests a direct correlation between the number of eosinophils in the sputum to the airway hyperresponsiveness of an individual. Eosinophils also release several eosinophil-derived mediators such as transforming growth factor-beta (TGF-β) while modulating Th2 responses [91]. Therefore, IL-13 is a biomarker for Th2-derived bronchial asthma and is upregulated in asthmatic airways [92]. Although the least abundant white blood cell in the lungs, eosinophils are a key diagnostic marker for the development of allergic airway diseases [93]. Eosinophils have also been found in larger numbers in asthmatic airways of patients who died within the sudden onset of an asthma attack compared to patients with slow-onset fatal asthma [94].

A few studies have shown that females are predisposed to eosinophilic asthma compared to males, an effect that is abolished upon removing female sex hormones [17,95]. However, this effect is seemingly via non-classical activation of the female sex hormone receptors, as studies have shown a lack of estrogen and progesterone receptor expression in eosinophils [79]. The effects of estrogen-inducing eosinophilia in women are attributed to estrogen and progesterone’s ability to degranulate eosinophils [17]. Ovariectomized (OVX) animals supplemented with estrogen have significantly lower numbers of eosinophils in the lung compared with ovariectomized mice not supplemented with estrogen [95]. In OVA-induced Balb/c mice, progesterone aggravates eosinophilic asthma and airway hyperresponsiveness. On the other hand, testosterone significantly reduces eosinophilic survival and adhesiveness [96]. Studies from our group have shown an elevated eosinophil cell count in female compared to male and OVX female mice, suggesting an important role of sex steroids, namely estrogen and testosterone, in lung function [20]. However, the direct effect of androgens on eosinophilic asthma is limited and requires further investigation.

### 4.3. Sex Steroids and Neutrophils

Neutrophils fall under the class of polymorphonuclear leukocytes that act as the first line of defense against bacterial and fungal infections and play an important role in the nonspecific immune system. Their inflammatory role was once thought to be restricted to phagocytosis and the release of enzymes and other cytotoxic agents, but it is now acknowledged that neutrophils also release inflammatory mediators that affect asthmatic airways [35]. Neutrophilic inflammation is observed during asthma exacerbations and, importantly, in subgroups of patients with severe asthma that are more steroid-refractory [97]. While neutrophils play an important role in pathogen elimination, persistent neutrophilia and the associated secretion of proteases have detrimental consequences in the airways, including airway injury and obstruction, mucus hypersecretion and airway remodeling [97,98]. Corticosteroids, the mainstay of asthma therapy, fail to suppress neutrophilic inflammation and may even promote neutrophil survival [99,100]. Therefore, neutrophils remain an important biomarker of asthma. Activated neutrophils produce several chemokines including interleukin-1 (IL-1), interleukin-3 (IL-3), interleukin-6 (IL-6), interleukin-8 (IL-8), TNF-α, interleukin-12 (IL-12), interferon gamma (IFN-γ), GM-CSF, and TGF-β. The surface-expressed lactoferrin released from neutrophils after contact with autologous CD4+ T cells has been shown to suppress Th1 cytokine levels, but with a tendency to enhance Th2 cytokine production [94]. Elevated neutrophil levels have also been found in the submucosal glands of patients with asthma and fatal-onset asthma compared to healthy controls [94,98]. Recently, the process of NETosis caused due to formation of neutrophil extracellular traps (NETs or ETs) has been identified in asthma. For instance, NETs have been detected in the airways of asthmatic patients [101,102]. Similarly, another group also reported the presence of NETs in BAL samples of neutrophilic asthma patients [103].

Despite the essential role of neutrophils in the inflammatory response, few studies investigate the effects of sex steroids on neutrophils. Generally, a neutrophilic sexual dimorphism exists between males and females post-maturity, with females exhibiting a significantly higher neutrophil count than males [104]. This translates into a significantly lesser ability of males to produce NETs following allergic stimuli, suggesting an inhibitory effect of androgens on neutrophils [105]. Earlier studies demonstrated a significant difference in neutrophil counts between men and women, which was later observed to be due to cyclical variations in the menstrual cycle of females [104,106]. However, an increase in the neutrophil count and an increased production of NETs are observed during pregnancy, implying a critical role for female sex steroids [107]. The female sex steroids, estrogen and progesterone, were implicated since they modulate the immune system during the different reproductive phases [108]. In addition, studies done on women taking oral contraceptive therapies show no change in neutrophil counts, signifying no immune system interference with exogenously administered treatment strategies. Several studies have investigated the expression of ERα and ERβ subtypes in the neutrophils of premenopausal women from different menstrual cycle phases [109]. In vitro incubation of neutrophils from women in the follicular phase with 17β-estradiol (E_2_) upregulated ERα and ERβ subtypes [110]. However, although E_2_ causes an upregulation of both ER subtypes, the expression of ERα seems to be more pronounced in men. Androgen receptor (AR) seems to be highly expressed in neutrophils, modulating various androgen (testosterone and DHT) mediated signaling mechanisms [111,112,113]. Likewise, PR is also present in neutrophils, with various studies postulating progesterone’s delayed apoptotic effects on neutrophils [108]. Previous studies have shown increased neutrophil count in females compared to males in an ozone-induced lung inflammation model [54]. Studies utilizing an allergic mouse model of asthma showed reduced neutrophilic inflammation upon treatment with testosterone [114]. Since neutrophils constitute the most abundant type of granulocyte in the immune system, studies have examined the effect of estrogen and progesterone on neutrophilic degranulation and activation. Incubation of neutrophils with estrogen and progesterone reduces the release of granular contents from neutrophils as measured by the release of beta-glucuronidase and lysozyme [115]. With respect to neutrophilic inactivation, estrogen has an increased inhibitory effect on neutrophil activation compared to progesterone [116]. Furthermore, it is well known that severe asthmatics with increased neutrophil cell counts undergo IL-17-mediated inflammation [117,118]. Indeed, studies have reported increased IL-17 and Th17 cells in the bronchoalveolar lavage (BAL) fluid from patients with severe asthma [119,120]. Another study showed that estrogen and progesterone, in combination, upregulate the expression of IL-17A in Th17 immune cells via a Let-7f/IL-23R pathway [121]. Studies using testosterone as an adjuvant on human neutrophils have shown increased phagocytic activity, suggesting a potential improvement in cell viability and an overall protective role of testosterone [122]. Pregnancy is associated with increased neutrophil count due to a unique challenge for the maternal immune system from holding her semi-allogenic fetus [108]. Studies performed on patients using oral contraceptive therapies also show increased neutrophil numbers [106]. Moreover, it is known that severe asthma showing increased neutrophil infiltration in both sexes is associated with IL-17 [34].

### 4.4. Sex Steroids and B Cells

B cells play the role of a central mediator in the pathophysiology of asthma. They are regarded as immune system promoters due to their innate ability to propagate antigen-specific antibodies. They are responsible for eliciting humoral responses against a variety of antigens. Regarding sex steroid receptor expression in B cells, ERα, ERβ, G-protein estrogen receptor (GPER), and the PR are all expressed [123]. Interestingly, the AR localizes to the nucleus and plasma membrane in B cells [124]. B cells can activate specific Th2 cells to produce numerous cytokines by presenting antigen fragments in combination with the major histocompatibility complex (MHC). This activity leads to further B cell activation and IgE release. These IgE antibodies bind to the high-affinity IgE receptor, Fc epsilon receptor I (FcεRI), that is present in mast cells, eosinophils and basophils, thereby sensitizing these cells to antigen exposure [125]. Males appear to be disproportionately more affected by asthma than females. However, following puberty, significantly more females report asthma symptoms [126,127]. This discrepancy suggests that estrogen may have a stimulatory function in antibody production, whereas androgens suppress antibody production. Other studies, although not in human pulmonary systems, have suggested that ovariectomy leads to an increase in the development of B cells in the bone marrow [128]. A few studies have postulated a concentration-dependent, pro-inflammatory effect of estrogen on IgE-mediated immunity and mast cell activation [129,130]. Further mechanistic insight into estrogen-dependent activation of IgE-mediated humoral immunity is needed. Pregnancy causes a reduction in the development of B lymphocytes and a decrease in the secretion of antibodies [131]. Testosterone has been associated with B-cell tolerance, suggesting an alternative mechanism by which androgens may modulate immunoglobulin production [132]. Furthermore, the administration of DHEA (a precursor of androgen) also suppresses airway inflammation associated with a Th2 inflammatory response [133]. Although the mechanism of action of DHEA is not fully elucidated, it is well known for its androgenic effects in various studies [134,135]. Specific studies of sex steroid effects on B cell lymphopoiesis are complex and not yet fully elucidated.

### 4.5. Sex Steroids and T Cells

The discovery of T cells and the supposed imbalance between mutually co-existing Th1 and Th2 cells sparked experimental observations in allergic diseases, including asthma [136,137]. However, this explanation of two sub-types of a T cell immune response proved to be overly simplified. Soon after, naturally occurring and adaptive Treg cells were discovered, which remain the major therapeutic intervention target of choice, capable of modulating Th1 and Th2-mediated immune responses in asthma [138,139,140]. In addition, Th1 and Th2 cytokine patterns are not the only two possible cascades during a Th cell effector response. A third subtype, known as the T helper 3 (Th3), has been described amongst immune responses [141]. Th1 cells produce IFN-γ, TNFα and IL-2. These cytokines evoke strong cell-mediated and phagocyte-dependent immune responses. Th2 cells are more diverse and constitute antibody responses by producing the IgE class of immune responses by evoking IL-4, IL-5, IL-6, IL-9, IL-10 and IL-13 [142]. Th3 responses mediate the production of TGF-β. Despite this complexity of the response, chronic asthma is particularly Th2 cell-driven. Until recent years, Th1, Th2 and Th3 were the main effector subsets of Th cells. However, a newer subset of the T cell, known as the Th17 cell, has emerged as an independent, yet essential Th subset. The Th17 cells secrete their main cytokine, IL-17, whose main function is neutrophilic recruitment through producing and releasing specific chemo-attractants such as CXCL8 [58]. We will review these cytokine patterns, emphasizing the sex steroid effects on the major cytokine responsible for regulating each Th cell subset. Typically, an antigen is presented to a dendritic cell, better known as the antigen-presenting cell, which initiates the differentiation of naïve T cells into their subsets depending on the stimuli received. As mentioned, effector Th cells are primed with CD4+ and segregate based on their distinct cytokine phenotype into distinct Th subset [142]. TNFα constitutes the “first wave” cytokine in the Th1 cell effector responses released on initial exposure of antigen-presenting cells. Both TNFα mRNA and protein are upregulated in asthmatic airways [143,144]. IL-13 is a cytokine of particular interest recently since mRNA and protein studies show increased IL-13 expression in asthmatic patients with a Th2 mediated response [145,146,147].

Sex steroids modulate several aspects of T cell differentiation and function. It is well established that females exhibit increased Th2 immune responsiveness compared to men [148]. Estrogens, in particular, favor the Th2 immune responses by suppressing the Th1 response [148]. However, other reports have suggested more pleiotropic roles of estrogens with a concentration-dependent estrogen effect facilitating induction of specific immune responses at lower concentrations while inducing immune suppression at higher concentrations, for example, during pregnancy. Progesterone is also known to favor the development of a Th2-type cytokine response by suppressing Th1 responses, thereby providing optimal conditions for maintenance of a pregnant state [149,150]. Testosterone, on the other hand, favors the Th1 immune response by reducing the production of Th2-mediated inflammatory cytokines [151]. Moreover, testosterone supplementation results in a higher Th1:Th2 ratio in men than women [152]. These observations suggest a protective role for sex steroids, especially testosterone in men, by activating the nonspecific immune system. As mentioned earlier, a shift to the Th2 response corresponds with clinical findings of increased incidence and severity of asthma in women post-puberty [148,153]. This suggests that sex differences in asthma might be due to sex differences in T cell-mediated cytokine production. However, ER expression in T cells remains controversial. Some studies suggest a lack of ER on T cells [154], while others indicate expression of ERα and GPER but not ERβ or PR in T lymphocytes [154,155]. Estrogen increases Th17 cell differentiation via an IL-23-mediated mechanism in mice and humans [121]. Studies have shown decreased lymphocyte infiltration and IL-17A-mediated airway inflammation in mice [156,157]. Given the lack of certainty regarding ER expression in T cells, it is possible that immunomodulatory effects of estrogen on T cells may be direct or mediated through other cells such as stromal cells, ASM, fibroblasts, or endothelial/epithelial cells, which display ERs, as well as antigen-presenting cells such as macrophages.

**Figure 3:**
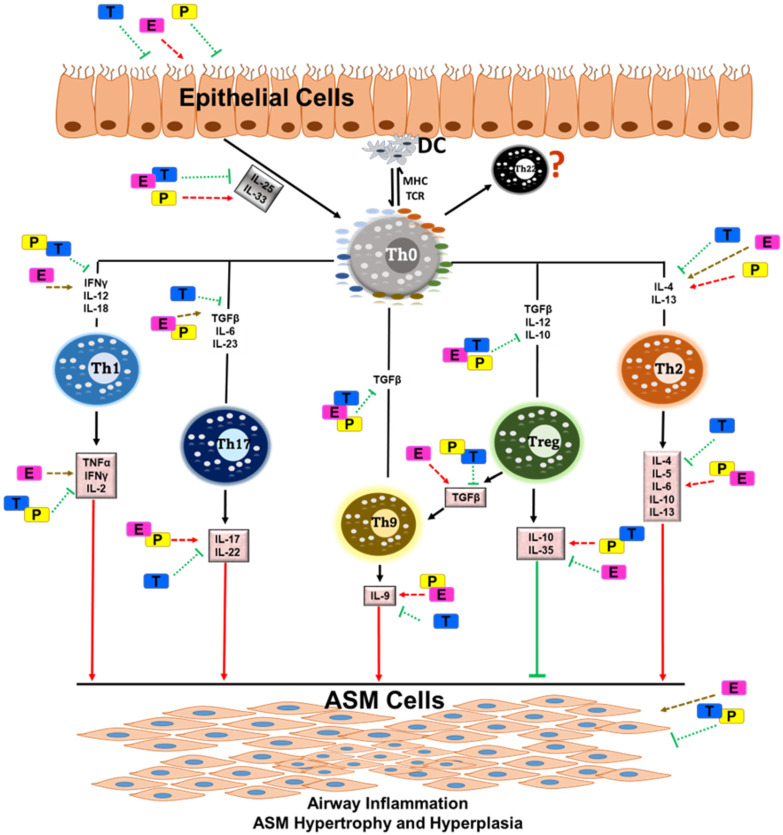
It is well known that asthma is a multifaceted respiratory disorder consisting of allergic triggers on the immune system and alterations in the structural cells of the airway. Overall, airway inflammation is mainly orchestrated by the T cell subsets, and the regulation of T cell subsets from naïve (Th0) cells may regulate cytokine release towards specific T-cell types. These T cells and their cytokine release are influenced by sex steroids such as estrogen, testosterone and progesterone. All these T cell subsets play key roles in recruiting, activating, and promoting the survival of multiple cell types along with altered pro- and anti-inflammatory cytokines in the airways, subsequently leading to airway smooth muscle cell (ASM) inflammation and remodeling. This is greatly influenced by circulating sex steroids, as discussed in this review. Pathways/cytokines aggravated in asthma are marked with red arrows (single and dotted), whereas pathways inhibiting airway inflammation in asthma are marked with green arrows (single and dotted). Yellow arrows denote unknown effects. Abbreviations: DC, dendritic cells; MHC, major histocompatibility complex; TCR, T-cell receptor; Th0, T helper cell (naïve); IFNϒ, interferon-gamma; IL, interleukin; TGF-β, transforming growth factor-beta; TNFα, tumor necrosis factor-alpha; E, estrogen; T, testosterone; P, progesterone.

On the other hand, ubiquitous expression of the AR was demonstrated in T lymphocytes [124]. In addition, it is known that testosterone reduces the total cell count of IL-13+ Th2 immune cells [151]. Several studies provide additional evidence to suggest a crucial role of testosterone signaling via AR in mitigating Th2-mediated allergic inflammation in the lung [158,159,160]. However, the underlying mechanism by which testosterone signaling provides an inherent advantage to males compared to females is unclear. An overview of sex steroid effects on T cell subsets is depicted in Figure 3.

### 4.6. Sex Steroids and Monocytes/Macrophages/Dendritic Cells (Mononuclear Phagocyte System)

The mononuclear phagocyte system represents a group of leucocytes derived from the bone-marrow-derived myeloid cells. These cells circulate in the blood as monocytes, which further localize in tissues as macrophages or dendritic cells [161]. Dendritic cells represent a specialized class of mononuclear phagocytes, functioning as highly efficient antigen-presenting cells (APCs), activating T cells for further Th1 or Th2 differentiation as described above. Although the sex steroid receptors such as ERα, ERβ, GPER and PR, as well as the AR, are present across the sexes in monocytes and macrophages, there is a complete absence of the female sex steroid receptors in dendritic cells, with dendritic cells showing abundant expression of the AR at both the nuclear and cytoplasmic levels [124]. A similar phenotypic differentiation is observed in the case of macrophages, which differentiate into M1/M2 polarization depending on inflammatory stimulus [162]. In allergic mouse models of asthma, estrogen via ERα signaling promotes a Th2-mediated immune response via M2 macrophage polarization [69], whereas testosterone, via AR signaling, attenuates Th2-mediated immune responses characterized by impaired M2 polarization and airway hyperresponsiveness [20,63]. Inflammatory toll-like receptor-mediated responses are also enhanced by estrogen and reduced by testosterone exposure in respiratory virus syncytial infection [163]. Progesterone treatment increases monocyte numbers but reduces functional activities of pro-inflammatory cytokines such as IL-12 and TNFα [86]. Additional studies to determine the exact role of progesterone on the mononuclear phagocyte system in asthma are required.

### 4.7. Sex Steroids and Natural Killer Cells

Natural killer (NK) cells constitute 10% of the lymphocytic population in the lung and blood. NK cells play a regulatory role in the body by interacting with other immune cells through binding with multiple receptors on the surface of the target cells. NK cells exhibit the presence of ERα, ERβ and the PR but lack GPER and AR [124]. Since the lung is the major organ affected by respiratory infection and inflammation, NK cells are known to control chronic inflammation, as seen in asthmatic airways, by stimulating the early production of IFN-γ [164]. Recent studies have indicated a higher number of NK cells in males than females. Interestingly, this difference was reversed in old age [165]. In females, NK cell counts also varied with stages of the menstrual cycle and pregnancy, implicating a role of female sex steroids in NK cell regulation [37]. Interestingly, a recent study demonstrated that estrogen increases mRNA levels of proteins in non-small cell lung cancer cell lines. Shedding of these proteins impairs the anti-tumor immunity elicited by NK cells, resulting in reduced anti-tumor responses [166]. Despite the potent antiviral effect of NK cells against pathogens, the known effects of independent sex steroids on NK cell activity in the lungs are very limited [167].

### 4.8. Sex Steroids and Mast Cells

Mast cells (MCs) constitute another important tissue-based cell that orchestrates the migration of inflammatory cytokines. Apart from T cells, airway MCs are also an important source of various vasoactive pro-inflammatory mediators such as histamine, serotonin, TNFα and IL-4 [168]. Many studies reported the expression of ERα (not ERβ), and both PRs reported in MCs [169]. MCs are also popularly addressed as immediate hypersensitivity effector immune cell types and are widely located in the respiratory tract [170]. In vitro studies have found that the physiological concentration of estrogen via ERα triggers a rapid influx of extracellular Ca^2+^ in MCs, which could subsequently lead to airway contraction and hyperresponsiveness [130]. Studies have shown that physiological concentrations of estrogen induce MC degranulation and enhanced release of cytokine mediators such as histamine, prostaglandin, leukotrienes and several other cytokines [77,171]. A separate study observed a reduction in MC numbers and histamine secretion upon ovariectomy [77]. MCs have also been shown to express the AR [172]. However, in the same study, estrogen and testosterone treatment did not affect MC degranulation [172]. In contrast, studies have also shown that testosterone stimulates MC degranulation and enhances histamine release. Conversely, progesterone abrogates MC degranulation and inhibits histamine release in a study performed on rat peritoneal MCs [173]. In a separate study, administration of an ER antagonist in control female rats in MCs reduced the progress of allergic airway disease [169,174]. Interestingly, another study to check the interdependent effect of sex steroids on activation of MCs was performed in female rats, and it was found that estrogen treatment stimulated activation of peritoneal MCs by the release of histamine, whereas concurrent administration of progesterone, testosterone, or DHT abrogated the release of histamine [169,175].

### 4.9. Treg: A Window of Opportunity?

Although asthma is characterized as an eosinophilic inflammatory and Th2-dependent disorder, newer evidence suggests a relationship between asthma and the heterogeneity of T cells [8,176]. As already mentioned, Th1-mediated immune responses are known to be responsible for the generation of organ-specific autoimmunity, while Th2-mediated responses are critical for allergic inflammation. T cells expressing cytokines of both patterns are naïve T cells or designated as type 0 or Th0. Soon after the initial description of Th1/Th2 associated cytokines, an array of different T cells appeared depending on their cytokine patterns with the advent of different CD4+ T cells, such as Th17, Th22, Th9 and Treg cells [9,177,178]. Th17 cells produce IL-17 and IL-22, which are stimulated in the presence of IL-23. Th9 is an IL-9-producing T-cell subset induced upon TGF-β stimulation [8]. Recently, a new subset of human T cells called Th22 was identified. However, no detailed studies are reported to date that verify Th22 existence in the airway [9].

With the rapid progress of research in the field of Tregs, it is clear that these cells have a crucial controlling role in airway diseases [9]. The ability of these cells to exert immunosuppressive actions and dampen immune responses against inflammatory triggers is well established in various disease models [178,179]. Therefore, Tregs are an important yet unexploited T-cell subset whose behavior might be targeted to attenuate clinical symptoms of airway diseases [180]. Typically, when an antigen stimulates a Th0 cell, it promotes differentiation into Tregs. Tregs function as good “immune” cells by maintaining the immune response triggered by Th2 cells within a normal range [60]. In this way, the Tregs regulate immunotolerance and control airway hyperresponsiveness by suppressing Th2-mediated immune responses to allergens. This effect of Treg cells is predominantly due to their secretion of suppressive cytokines such as TGF-β, IL-10, IL-35 and other cell-lysing molecules [178]. When dysfunctional Tregs fail to suppress aggravated Th2 responses, asthma or other allergic/inflammatory diseases develop [181]. It is noteworthy to mention that studies clarifying the role of Tregs in asthma have found that, in humans, IL-10 and TGF-β released by Tregs significantly suppress airway inflammation [182,183,184,185,186]. A study of exogenous estrogen administration in Treg-depleted mouse models reported that E_2_ via ERβ was required to control pro-inflammatory responses in pneumonia. In the same study, E_2_-treated Tregs via ERβ and not ERα rescued pneumonia-induced lung injury [187]. This aligns with our published studies where ERβ agonist administration significantly improves airway compliance in vivo in asthmatic mouse models [188]. Consistent with this study, another report on female asthmatics showed improved asthmatic symptoms in females on oral contraceptives compared to control female asthmatics [189]. Similarly, DHT via AR signaling increased Treg suppressive function by inhibiting an ST2+ Tregs (Tregs promoting Th2 response) mechanism [190].

The incidence of asthma is highest during the reproductive years of a female, at a time when the sex steroids are at their peak levels [2,14]. Moreover, Tregs fluctuate throughout a female’s reproductive cycle or pregnancy states, as with age [191]. Even though the number of Tregs increases with age, the functionality of the overall immune system decreases [192]. In addition, clinical evidence of reduced Tregs is observed in elderly asthmatic patients [193]. Thus, sufficient evidence indicates that Tregs provide a natural immune tolerance against asthma and strategies to exploit Treg function represent exciting therapeutic possibilities.

## 5. Sex Steroids and Structural Cells of the Airway

ASM cells are the major contractile machinery involved in multiple cellular processes in the lung. Increased smooth muscle mass and other fibrotic changes and inflammation contribute to asthma symptoms [5,7]. Considerable investigations on the role of estrogen and its differential capacity of activating ERα and ERβ on ASM cells have been carried out [194,195]. These data suggest that estrogen plays a significant role in the regulation and function of the ASM cell during inflammation or asthma. Limited studies have investigated the role of progesterone in airway function, particularly in asthma [196]. Additionally, a clear role of testosterone needs to be elucidated in airway function and asthma, given that testosterone has a protective role in the ASM cell [20]. While some studies associate estrogens with an asthma-instigating role, we and others demonstrate estrogen having a mitigating effect on asthma via differential ER activation, particularly via ERβ [194,196,197,198,199,200]. These uncertainties of a clear role of sex steroids in asthma warrant consideration of mechanisms independent or/dependent on sex steroids. In this regard, kisspeptins (Kp) are a crucial yet understudied peptide that could play an important role in asthma and inflammation [201]. With several reports studying the expression and function of Kp and its receptor, KISS1R, in cancer and other disease models, our study showed, for the first time, robust expression and function of Kp/KISS1R in human ASM [201]. We showed differential expression of ASM Kp/KISS1R, with asthmatics showing the lowest expression of Kp and KISS1R. Moreover, we also demonstrated an anti-proliferative effect of Kp-10 by inhibiting PDGF-induced ASM cell proliferation in non-asthmatic and asthmatic human ASM [201]. The role of estrogen vs. testosterone in the regulation of Kp is not known and needs to be elucidated to determine any sex steroid signaling crosstalk responsible for sex differences in asthma. Epithelial cells of the airway or the “initiator cells” propagate the inflammatory process in airway inflammation and remodeling. However, multiple studies have found that androgens exert an epithelium-independent bronchial relaxation by blocking voltage-gated channels [202]. Another study suggests an inhibitory effect of DHEA on the TGF-β-induced epithelial to mesenchymal transition in bronchial epithelial cells [203]. Surprisingly, although present in the nasal epithelial lining, few studies have clarified the functional effect of progesterone on epithelial cells. One study showed reduced ciliary motility by progesterone, an effect ameliorated upon the addition of estrogen [204]. It is well established that estrogen facilitates activation of epithelial origin nitric oxide, which upon inflammatory triggers, is increased [148]. Overall, there is limiting and conflicting data on the effects of sex steroids on the bronchial epithelial cells in asthma. Other studies have also shown that ovariectomy in rats leads to pulmonary fibrosis, while supplementation using 2ME significantly reverses the fibrotic phenotype [205]. Similarly, in a bleomycin-induced lung fibrosis model, castration restores normal lung function, whereas DHT replacement therapy worsens it [206].

Fortunately, understanding the mechanisms by which sex steroids influence structural cells of the airways has garnered new interest with novel findings from researchers working on ASM, epithelial cells and fibroblast in the lungs [5,39,201,207,208]. Sex steroids influence structural cells of the airways and immune cells independently. However, we ask the following questions: (1) Do sex steroids modulate structural cells of the airway via effects on immune cells? (2) Do direct effects of sex steroids on airway structural cells, in turn, modulate the fate of immune cells? In either question, estrogen, progesterone, or testosterone are likely to directly alter gene regulation via nuclear activation resulting in altered airway reactivity and differences in asthma. Understanding sex steroid modulation of immune cell regulation of airway structural cells in asthma is a major deficit in the field.

## 6. Coronavirus Disease: What’s Sex/Sex Steroids Got to Do with It?

SARS-CoV-2 is the novel coronavirus responsible for COVID-19 that created a global pandemic in November 2019, starting in Wuhan, China. On 11 March 2020, the World Health Organization (WHO) declared the COVID-19 pandemic, affecting almost 499 million people globally and counting [209]. Several reports indicate that asthmatic individuals are at a higher risk of acquiring SARS-CoV-2 infection [210,211]. Moreover, adults with severe asthma show increased risks of adverse COVID-19 outcomes, including hospitalization, admissions to intensive care units (ICUs) and subsequent death [212]. Most importantly, several studies, including ours, have shown a possible influence of sex and sex steroids on the outcome of COVID-19 [208]. According to the Sex, Gender and COVID-19 Project 2020, agencies worldwide combined data on COVID-19 and reported a significant male predominance in COVID-19 morbidity and mortality [209]. An article collating sex-classified statistics showed that men were twice as likely to die from COVID-19 than women [213,214]. The mechanistic and physiologic basis for this observed difference is still under investigation. Some hypotheses, including ours, implicate sex steroids in differential effects of COVID-19. For example, we observed higher baseline expression of the ACE2 receptor in the ASM in males compared to females [208,215]. More importantly, when age-matched male and female ASM cells were exposed to physiological concentrations of the sex steroids independently, testosterone increased ACE2 expression, whereas estrogen downregulated ACE2 in ASM [208]. We believe that other structural cells of the lungs may be influenced by the cyclical variations of the intrinsic hormones. In almost all respiratory diseases, sex-specific immune responses have been postulated, which is also true for COVID-19, which shows prominent sex differences in the immune response against the viral pathogen [216]. We also accept that the immune response of each sex may play an integral part in ACE2 biology. Much attention in recent times has also been paid to herd immunity, which is characteristic of certain geographical areas. Finally, we recognize that comorbidities such as age, lifestyle habits, smoking, hygiene, social and other associated factors also contribute to COVID-19 infection incidence and severity [217]. Therefore, an analysis of the role of these components in the sex-skewed frequency and mortality of COVID-19 needs to be performed.

## 7. Conclusions and Future Scope

The phenomenon of a gender switch in asthma is well established and has led to the hypothesis that sex steroids play a major role in asthma pathophysiology. Sex steroid signaling affects almost every cell of the organ system, including the immune cell system. Within the past few decades, there has been increasing recognition that cytokines and chemokines play an instrumental role in perpetuating the underlying mechanism of asthma progression. Newer translation research has unraveled the therapeutic effectiveness of alternative options targeting several cytokine receptors. These novel developing medications have shown promise in clinical investigations and might be especially effective in selective patients with multiple endotypes. Therefore, novel therapies for asthma may potentially lie in targeting the sex steroids and the immune system rather than focusing on a single sex-based therapeutic strategy or global immunosuppression.

Given the heterogeneity and wide spectrum of asthma, the standard biologicals investigated have been disappointing and still need improvement. Similarly, a prototype medicine recognizing a single target will apply to only a small patient pool out of the vast population that shows different endotypes of asthma. We are now entering an era of individualized medicine, which requires a clear understanding of our therapeutic interventions to account for the large, heterogeneous patient populations widely seen in asthma. Future mechanistic studies involving the role of sex steroids and key hormonal signaling pathways and their interactions with other cell systems will help to identify novel therapeutic targets and key asthma endotypes, thereby providing evidence for a more personalized and precision-based asthma management strategy. The foundation of personalized or individualized medicine is to utilize the unique contributions of our biology and sex, including our immune system, to provide and curate novel targets for more effective treatment therapies for asthma management. This review aims to summarize the studies that support the non-gonadal role of sex hormones and their biological influences on several aspects of the immune system in the context of respiratory biology. Looking ahead, more studies are required to understand immune cell behavior responsible for yet under-studied sex-based differences in asthma. Future work should provide appropriate immunological, structural, cellular and pharmacological models to encompass the intricate pathobiological networks underlying asthma.

## Data Availability

Not applicable.
